# Effect of Gallic acid in potentiating chemotherapeutic effect of Paclitaxel in HeLa cervical cancer cells

**DOI:** 10.1186/s12935-019-0868-0

**Published:** 2019-06-03

**Authors:** Nora M. Aborehab, Nada Osama

**Affiliations:** 1 Department of Biochemistry, Faculty of Pharmacy, October University for Modern Sciences and Arts (MSA University), Giza, 12611 Egypt; 2 Department of Biochemistry, Faculty of Pharmacy, Menoufia University, Menoufia, 32511 Egypt

**Keywords:** HeLa cells, Gallic acid, Paclitaxel, P53, Caspase 3

## Abstract

**Background:**

Cervical cancer is the fourth most common cancer affecting women worldwide. Paclitaxel/Carboplatin is one of the most commonly prescribed regimens in cervical cancer treatment. Although chemotherapeutic drugs are very effective, severe side effects and development of drug resistance limits the use of these drugs. The use of natural products with anticancer activity may help to partially overcome these issues.

**Methods:**

In the present study, we investigated the ability of Gallic acid, to potentiate the anti-cancer effects of Paclitaxel, Carboplatin and Paclitaxel/Carboplatin combination in human HeLa cells by performing MTT assay, cell cycle analysis and RT-PCR assay and Western blotting for some apoptotic markers.

**Results:**

Our results revealed that the highest cytotoxic effect, the highest induction of apoptosis and significant elevation in P53 and Caspase 3 levels was seen in Paclitaxel/Gallic acid combination.

**Conclusion:**

These results indicate that Gallic acid potentiates Paclitaxel effect and that Paclitaxel/Gallic acid combination could represent a promising alternative with lower side effects-for Paclitaxel/Carboplatin combination in treatment of cervical cancer treatment.

## Background

Cancer is the second leading cause of death globally, and is responsible for an estimated 9.6 million deaths in 2018 [1]. Lung cancer is the most frequent cancer and the leading cause of cancer death among males, followed by prostate and colorectal cancer (for incidence) and liver and stomach cancer (for mortality). Among females, breast cancer is the most commonly diagnosed cancer and the leading cause of cancer death, followed by colorectal and lung cancer (for incidence), and vice versa (for mortality). Cervical cancer ranks fourth cancer type, for both incidence and mortality, affecting women worldwide [2]. Treatment of cancer with a single therapy is rarely effective as cancer development involves many features of the cell [3]. Combination therapy is a prominent approach in cancer chemotherapy. The toxicity is significantly less because different pathways will be targeted and lower dosage of each individual drug is required [4, 5].

Taxane and platinum-based combination chemotherapy is the treatment of choice for ovarian cancer [6]. Platinum-based drugs exerts cytotoxicity mainly through the formation of DNA adducts leading to hindering of DNA replication and induction of apoptosis, while Taxanes target microtubules leading to suppression of spindle-microtubule dynamics, which results in cell cycle arrest and eventually, apoptosis [7, 8]. Upon diagnosis, most ovarian cancer patients are administered combination therapy containing dose-dense Taxanes, usually Paclitaxel, and a platinum agent, usually Carboplatin, as it causes much less cytotoxicity and nephrotoxicity compared with Cisplatin as the platinum agent [9].

It has been proposed that cell cycle arrest induced by Paclitaxel possibly inhibits DNA repair leading to accumulation of Carboplatin-DNA adducts and enhances the antitumor activity [10].

However, the use of many chemically synthesized anticancer drugs has caused considerable side effects to patients. Therefore, the discovery and development of new drugs based on natural products have been the focus of much research [11, 12].

Some reports have shown to indicate the use of natural products in suppressing cancer proliferation via different mechanisms in the treatment of different types of cancer as breast cancer, head and neck cancer cell [13–15].

Gallic acid (GA) is a polyhydroxyphenolic compound which can be found in various natural products, such as green tea, grapes, strawberries, bananas and many other fruits [16]. A number of studies have demonstrated the potential anticancer activity of Gallic acid and its derivatives, both in vivo and in vitro [17, 18]. In fact, anti-cancer activity of Gallic acid has been reported in various cancer cells, including human ovarian cancer cells [19–22]. It was proven that the anticancer effect of Gallic acid is due to its ability to inhibit cell proliferation and to induce apoptosis [19, 23, 24].

In this study, we aimed to investigate the possible synergistic or antagonistic effect of Gallic acid with Paclitaxel, Carboplatin, Paclitaxel/Carboplatin combination therapy.

## Materials and methods

To compare the anticancer effects of Paclitaxel, Carboplatin and Gallic acid individually and in-combination on growth and apoptosis in HeLa cells, Growth inhibition assay, cell cycle analysis, RT-PCR assay and Western blot analysis for some apoptotic markers were performed.

### Reagents

Paclitaxel (Taxol^®^, 6 mg/ml, Bristol-Myers Squibb, Egypt) and Carboplatin (Carboplatin^®^10 mg/ml, MYLAN, France) were purchased from Egyptian market.

Gallic acid was purchased from Sigma Aldrich, USA. 3-(4, 5-dimethylthiazol-2-yl)-2,5-diphenyltetrazolium bromide dye was purchased from BIO BASIC, Canada. An Annexin V-fluorescein isothiocyanate (FITC)/Propidium iodide (PI) double staining kit was purchased from BioVision Research Products, USA. A RNeasy Mini Kit was purchased from QIAGEN, Venlo, Netherlands and a One-Step RT-PCR Kit was purchased from Bio-Rad, California, USA. Primary and secondary antibodies for western blotting were purchased from Cell Signaling Technology, Massachusetts, USA and Danko, California, USA, respectively. Materials and chemicals for electrophoresis were obtained from Bio-Rad (California, USA), Sigma Aldrich, USA and Roche, Basel, Switzerland.

### Cell culture

The HeLa cells, a human cervical cancer HeLa cell line, were supplied by VACSERA (Giza, Egypt). The cells were cultured in RPMI-1640 medium (Lonza, Switzerland) supplemented with 10% fetal bovine serum (Gibco), 1% penicillin and 1% streptomycin (Sigma Aldrich, USA) at 37 °C under an atmosphere of 5% CO_2_ and 95% air.

### Growth inhibition assay

The cell growth inhibition effects of serial dilutions of Paclitaxel (600, 300, 150, 75, 37.5, 18.75, 9.37, 4.68, 2.34, 1.17 µg/ml), Carboplatin (1000, 500, 250, 125, 62.5, 31.25, 15.62, 7.81 µg/ml), Gallic acid (10,000, 5000, 2500, 1250, 625, 312.51 µg/ml) and combinations of various treatment were detected by 3-(4,5-dimethylthiazol-2-yl)-2,5-diphenyltetrazolium bromide dye (MTT) assay [25]. Drug combinations and concentrations tested are shown in Table 1.

**Table 1:** The tested drug combinations

Mixture	Drugs and its concentration
Mix 1	IC50 of Paclitaxel (112.5 µg/ml) + IC50 of Carboplatin (323.5 µg/ml)
Mix 2	IC50 of Paclitaxel (112.5 µg/ml) + MNTC of Gallic acid (312.5 µg/ml)
Mix 3	IC50 of Carboplatin (323.5 µg/ml) + MNTC of Gallic acid (312.5 µg/ml)
Mix 4	IC50 of Paclitaxel (112.5 µg/ml) + IC50 of Carboplatin (323.5 µg/ml) + MNTC of Gallic acid (312.5 µg/ml)
Mix 5	1/10 from IC50 of Paclitaxel (11.2 µg/ml) + 1/10 from IC50 of Carboplatin (32.3 µg/ml)
Mix 6	1/10 from IC50 of Paclitaxel (11.2 µg/ml) + MNTC of Gallic acid (312.5 µg/ml)
Mix 7	1/10 from IC50 of Carboplatin (32.3 µg/ml) + MNTC of Gallic acid (312.5 µg/ml)
Mix 8	1/10 from IC50 of Paclitaxel (11.2 µg/ml) + 1/10 from IC50 of Carboplatin (32.3 µg/ml) + MNTC of Gallic acid (312.5 µg/ml)

Briefly, 1 × 10^5^ cells per well were seeded in 96-well microtiter plate and incubated at 37 °C for 24 h. to develop a complete monolayer sheet. After exposure to the indicated amounts of drugs for 24 h, medium was discarded and twenty microliters of MTT (BIO BASIC CANADA INC) solution (5 mg/ml in PBS) were added to each well of 96-well plate. The plate was placed on a shaking table, 150 rpm for 5 min, to thoroughly mix the MTT into the media then incubated for four additional hours at 37 °C and 5% CO_2_. Medium in plates was removed and 200 μl DMSO was added to each well to solubilize the formazan crystals (MTT metabolic product). Optical density was measured at 560 nm using a microplate reader (Mindray Mr-96A, China). Influences of tested compounds on HeLa cells proliferation were evaluated by the calculation of cell viability (%) using the following equation:

$${\text{Cell}}\;{\text{viability}}\;(\% ) = {\text{A}}_{560} \;{\text{of}}\;{\text{treated}}\;{\text{cells}}/{\text{A}}_{560} \;{\text{of}}\;{\text{control}}\;{\text{cell}} \times 100$$

In vitro cytotoxic activity was evaluated by the calculation of the concentration of the drug required to kill 50% of cells relative to the untreated cultures known as the half maximal inhibitory concentration (IC50). For Gallic acid the minimum dilution of Gallic acid with no toxic effect on the cells which corresponds to maximum non-toxic concentration (MNTC) was also detected.

### Cell cycle analysis and apoptosis assay

Annexin V-fluorescein isothiocyanate (FITC)/Propidium iodide (PI) double staining kit (BioVision Research Products, USA) was used to identify the cells in different phases. HeLa cells were treated with Paclitaxel (112.5 µg/ml), Carboplatin (323.5 µg/ml), Gallic acid (312.5 µg/ml), Mix. 1 (Paclitaxel (112.5 µg/ml) + Carboplatin (323.5 µg/ml), Mix. 2 (Paclitaxel (112.5 µg/ml) + Gallic acid (312.5 µg/ml), Mix 3 (Carboplatin (323.5 µg/ml) + Gallic acid (312.5 µg/ml), Mix 4 (Paclitaxel (112.5 µg/ml) + Carboplatin (323.5 µg/ml) + Gallic acid (312.5 µg/ml) for 24 h. After incubation, cells were harvested from the treated and normal samples. The cells were then resuspended in 500 μl of binding buffer, and then 5 μl of annexin V-FITC and 5 μl of PI were added in the dark for 5 min. The cell cycle distribution was then detected by flow cytometry (BD FACSCalibur, India).

### Real time quantitative PCR (RT-qPCR) assay for some apoptotic markers

HeLa cells were treated with selected doses of individual and combination drugs as mentioned previously in cell cycle analysis and incubated for 24 h. to perform mRNA expression analysis. Total RNA was then isolated from control HeLa cell and treated HeLa cells. RNA was extracted using RNeasy Mini Kits (QIAGEN, Venlo, Netherlands) according to the manufacturer’s protocol. RT-qPCR was performed by use of SYBR^®^ Green dye (iScriptTM One-Step RT-PCR Kit, Bio-Rad, California, United States) on Rotor-Gene Q real-time PCR cycler (QIAGEN, Venlo, Netherlands). The forward and reverse primers used for amplification of P53, Bcl-2, and Caspase 3 are listed in Table 2. Quantitative analysis was performed by the measurement of threshold cycle (CT) values during the exponential phase of amplification. ΔCT was calculated by the difference between the CT values of the P53, Bcl-2, Caspase-3 and the CT value of β-actin gene. Finally, fold changes of treated cells compared with untreated cells were calculated using the following equation: 2^ΔΔCT.

$$ \text{Where}\,\Delta \Delta {\text{CT = }}\Delta {\text{CTE}}{-}\Delta {\text{CTC}}$$

ΔCTE: the difference between the CT values of the P53, BCL-2, Caspase-3 and the CT value of B-actin gene in treated cells.

ΔCTC: the difference between the CT values of the P53, BCL-2, Caspase-3 and the CT value of B-actin gene in control HeLa cells.

**Table 2:** Forward and reverse primer PCR sequences for real-time PCR

Primer	Sequence of nucleotides (nt)	Size (nt)
P53		
Forward	5′-CCCCTCCTGGCCCCTGTCATCTTC-3′	24
Reverse	5′-GCAGCGCCTCACAACCTCCGTCAT-3′	24
Bcl-2		
Forward	5′-CCTGTG GAT GAC TGA GTA CC-3′	20
Reverse	5′-GAGACA GCC AGG AGA AAT CA-3′	20
Caspase 3		
Forward	5′-TTC ATT ATT CAG GCC TGC CGA GG-3′	23
Reverse	5′-TTC TGA CAG GCC ATG TCA TCC TCA-3′	24

### Western blot analysis

The expression of P53, Procaspase 3, Cleaved Caspase 3 proteins were evaluated using western plot analysis according to [26, 27]. In brief, HeLa cells were treated with DMSO (control), individual and combination drugs (mentioned previously in cell cycle analysis section) and incubated for 24 h. Then cells were then lysed in cold lysis buffer (100 mM NaCl, 10 mM Tris, 25 mM ethylenediaminetetraacetic acid (EDTA), 25 mM Ethylene glycol bis (2-aminoethyl) tetraacetic acid (EGTA), 1% (v/v) Triton X-100, 1% (v/v) NP-40 (pH 7.4), with 1:300 protease inhibitor cocktail (Sigma) and Phosphatase inhibitor cocktail Tablet (Roche). The cells were then immediately frozen at − 20 °C for 1 h for further lysis, and collected by cell scraper and sonicated 2 × 10 s. Protein concentration in the supernatant was measured with Bradford method. Equal amounts (20 µg) of total protein from control and treated cell extracts samples were mixed and boiled with SDS Loading buffer (750 mM Tris–HCl (pH 6.8); 600 mM dithiothreitol (DTT); 12% SDS; 0.012% Bromophenol blue; 60% glycerol) for 10 min, allowed to cool on ice and then loaded into SDS-polyacrylamide gel and separated by Cleaver electrophoresis unit (Cleaver, UK), transferred onto polyvinylidene fluoride (PVDF) membranes (BioRad) for 30 min using a Semi-dry Electroblotter (Biorad, USA) at 2.5 A and 25 V for 30 min. Membranes were blocked with 5% nonfat milk in Tris Buffered Saline Tween (TBS-T) for 2 h. at 37 °C and then incubated overnight at 4 °C with each primary antibody at indicated dilution (The primary antibodies against Caspase-3 antibody (1:1000, Cell Signaling Technology), Cleaved Caspase-3 antibody (1:750, Cell Signaling Technology), p53 (1:1000, Abcam), and β-actin (1:2000, Sigma). The blots were washed three times with TBS-T and incubated with secondary antibody (horse radish peroxidase-linked secondary antibodies (Dako) for another hour at room temperature, followed by washing for three times with TBS-T. Substrates were visualized by using chemiluminescent Western ECL substrate (Perkin Elmer, Waltham, MA) and analyzed using a CCD camera-based imager (Chemi Doc imager, Biorad, USA), and the bands intensities were then measured by ImageLab (Biorad). β-actin was used as the loading control.

### Statistical analysis

Data were analyzed using the GraphPad Prism 6.0 statistical program (GraphPad Software, Inc.), and statistical differences between groups were evaluated using two -way analysis of variance. P < 0.05 was considered to indicate a statistically significant difference.

## Results

### Growth inhibition assay

**Fig. 1:**
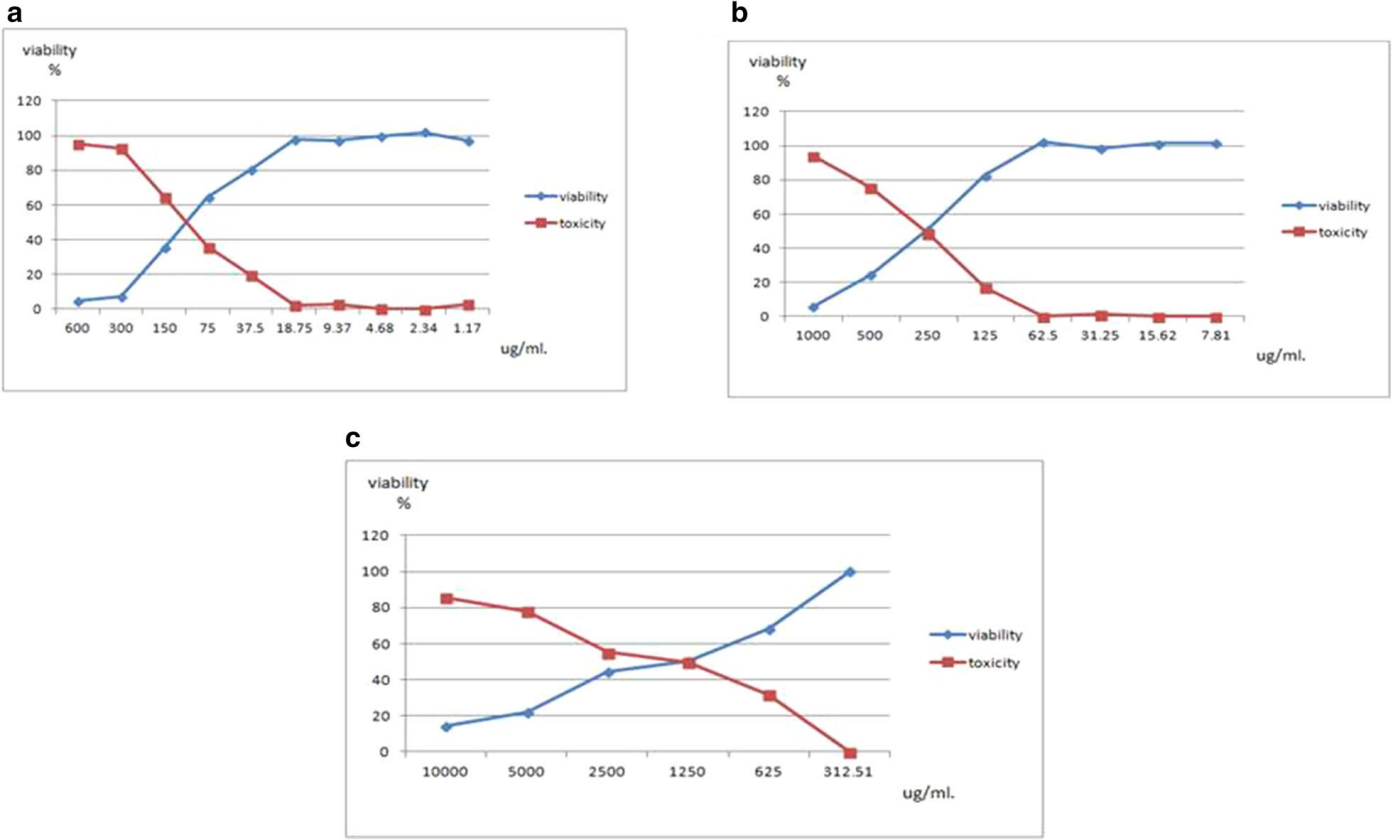
Effect of Paclitaxel (**a**), Carboplatin (**b**), and Gallic acid (**c**) on cell viability of HeLa cells at 24 h

The cytotoxic activities of Paclitaxel, Carboplatin, and Gallic acid were first assessed individually on HeLa cells via MTT assay (Table 3, Fig. 1). The three compounds showed significant inhibition of cell proliferation in a dose dependent manner. HeLa cells were most sensitive to Paclitaxel showing the IC50 of 112.53 µg/ml followed by Carboplatin that exhibited an IC50 of 323.57 µg/ml. The highest IC50 (1948.95 µg/ml) was recorded for Gallic acid. Also the MNTC for Gallic acid (312.51 µg/ml). These results reflect the lower cytotoxicity of Gallic acid when used on its own.

**Table 3:** Effect of individual drugs and combinations on cell viability of HeLa cells after 24 h

ID	Conc. µg/ml	Mean O.D	ST.E	Viability %	Toxicity %	IC50
HeLa	–	0.183	0.006351	100	0	µg/ml
Paclitaxel	600	0.009	0.000577	4.918033	95.08196721	112.53
	300	0.013333	0.000882	7.285974	92.7140255	
	150	0.064667	0.004485	35.33698	64.66302368	
	75	0.117333	0.002186	64.11658	35.88342441	
	37.5	0.147333	0.00318	80.51002	19.48998179	
	18.75	0.179	0.001528	97.81421	2.18579235	
	9.37	0.177667	0.002963	97.08561	2.9143898	
	4.68	0.182	0.003055	99.45355	0.546448087	
	2.34	0.186333	0.003844	101.8215	0	
	1.17	0.178	0.002646	97.26776	2.732240437	
Carboplatin	1000	0.011333	0.000333	6.193078	93.80692168	323.57
	500	0.045333	0.006333	24.77231	75.2276867	
	250	0.094	0.002517	51.36612	48.63387978	
	125	0.151667	0.005044	82.87796	17.12204007	
	62.5	0.187	0.003	102.1858	0	
	31.25	0.180667	0.000882	98.72495	1.275045537	
	15.62	0.185667	0.002186	101.4572	0	
	7.81	0.186	0.007506	101.6393	0	
Gallic acid	10,000	0.026667	0.00786	14.57195	85.428051	1948.95
	5000	0.04	0.006658	21.85792	78.1420765	
	2500	0.082	0.002309	44.80874	55.19125683	
	1250	0.092	0.001528	50.27322	49.72677596	
	625	0.125	0.005508	68.30601	31.69398907	
	312.51	0.183	0.003786	100	0	
Drugs combinations	HeLa	0.192667	0.003756	100	0	
	Mix 1	0.033	0.005196	17.12773	82.87226865	
	Mix 2	0.030333	0.003383	15.74367	84.25632775	
	Mix 3	0.051333	0.00348	26.64314	73.35686234	
	Mix 4	0.030333	0.003844	15.74367	84.25632775	
	Mix 5	0.056	0.001155	29.06524	70.93475891	
	Mix 6	0.051	0.005686	26.47013	73.52986973	
	Mix 7	0.037333	0.003528	19.37683	80.62317261	
	Mix 8	0.035333	0.004177	18.33878	81.66121693

Regarding the combination treatment, the highest cytotoxicity where seen in Mix. 2 (IC50 of Paclitaxel (112.5 µg/ml) + MNTC of Gallic acid (312.5 µg/ml) and Mix 4 (IC50 of Paclitaxel (112.5 µg/ml) + IC50 of Carboplatin (323.5 µg/ml) + MNTC of Gallic acid (312.5 µg/ml) which showed an 84.26% inhibition of cell viability. Interestingly, 81.66%, 80.62% and 73.53% inhibition of cell viability were noted, respectively in Mix. 7 (1/10 from IC50 of Carboplatin (32.3 µg/ml) + MNTC of Gallic acid (312.5 µg/ml) and Mix. 8 (1/10 from IC50 of Paclitaxel (11.2 µg/ml) + 1/10 from IC50 of Carboplatin (32.3 µg/ml) + MNTC of Gallic acid (312.5 µg/ml) and Mix 6 (1/10 from IC50 of Paclitaxel (11.2 µg/ml) + MNTC of Gallic acid (312.5 µg/ml).

Mix 6, 7 and 8, the concentration of treatments used was 1/10 of IC50 of Paclitaxel, Carboplatin and MNTC of Gallic acid; these combinations showed high cell cytotoxicity claiming for the potentiation of Gallic acid with Paclitaxel and Carboplatin even in low doses.

### Cell cycle progression

**Fig. 2:**
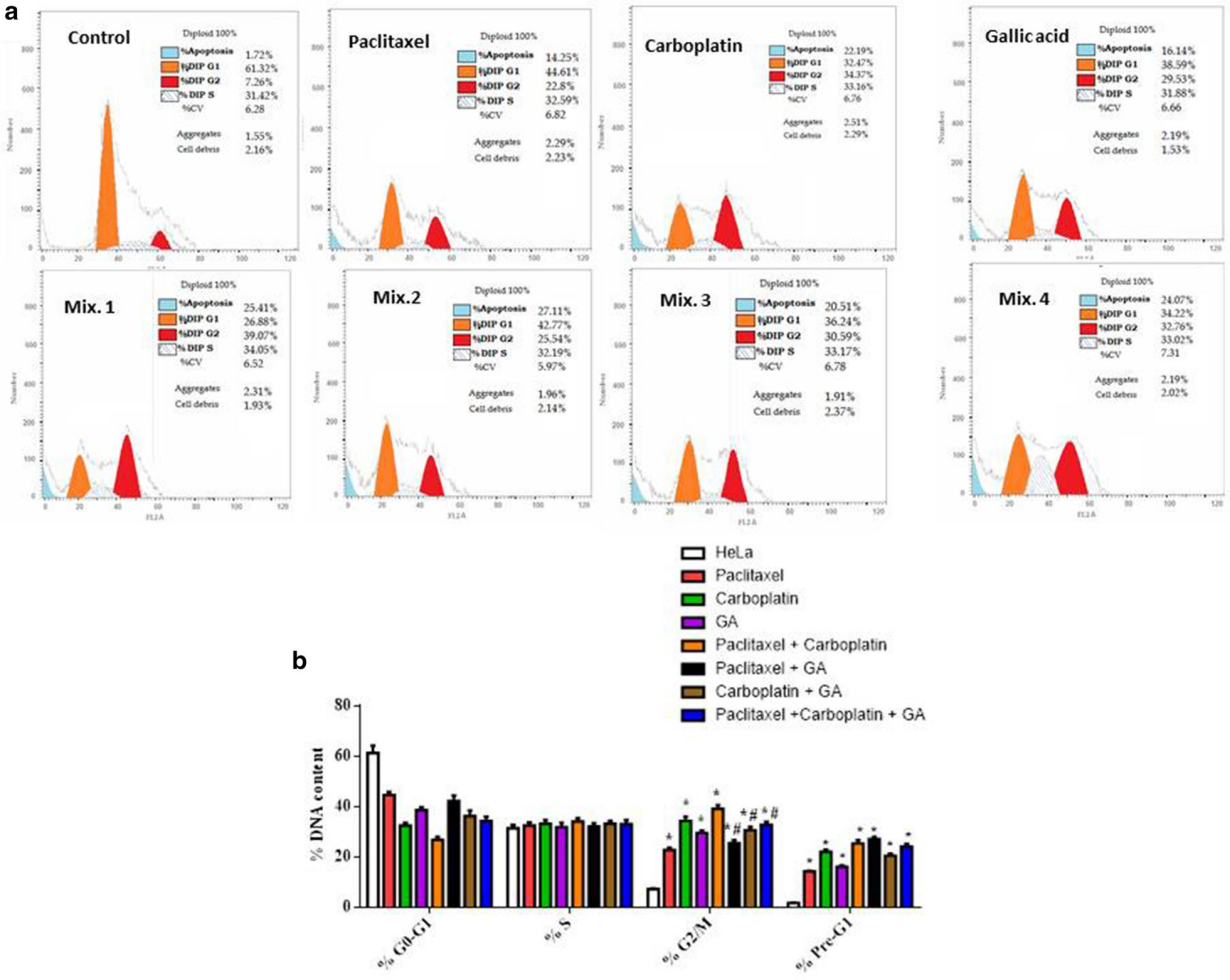
Cell cycle analysis of HeLa cells following treatment with different drugs. **a** Histogram showing the percentages of cells in different cell cycle phases. **b** Bar chart showing the percentages of cells in different cell cycle phases. *Significant from control group at P < 0.0001, ^#^significant from Paclitaxel + Carboplatin group at P < 0.0001; *GA* Gallic acid

The cell-cycle distribution and cell death, including apoptosis was analyzed using Annexin V-FITC/PI staining and flow cytometry. It was determined how each individual drug and the mentioned drug combinations changed cell cycle distribution and affected the anti-tumour activity (Table 4, Fig. 2).

**Table 4:** Cell cycle analysis of HeLa cells following treatment with different drugs

Sample data	Results			
ID	%G0–G1	%S	%G2/M	%Pre-G1
Cont. (HeLa)	61.32 ± 2.95	31.42 ± 1.34	7.26 ± 0.23	1.72 ± 0.02
Paclitaxel	44.61 ± 1.29	32.59 ± 1.23	22.8 ± 0.84	14.25 ± 0.32
Carboplatin	32.47 ± 1.09	33.16 ± 1.52	34.37 ± 1.72	22.19 ± 0.77
Gallic acid	38.59 ± 1.28	31.88 ± 1.76	29.53 ± 0.92	16.14 ± 0.63
Mix. 1	26.88 ± 1.22	34.05 ± 1.39	39.07 ± 1.49	25.41 ± 1.27
Mix. 2	42.27 ± 2.17	32.19 ± 1.31	25.54 ± 1.12	27.11 ± 0.92
Mix. 3	36.24 ± 2.19	33.17 ± 1.27	30.59 ± 1.38	20.51 ± 0.78
Mix. 4	34.22 ± 1.76	33.02 ± 1.61	32.76 ± 1.17	24.07 ± 1.13

### Flow cytometry analysis showing induction of apoptosis by Gallic acid

Cell cycle analysis showed that treatment of HeLa cells with Paclitaxel, Carboplatin, Gallic acid and the mentioned drug combinations resulted in growth arrest at the G2/M phase and it further showed an increase in the cell population in pre G1 population, which could be indicative of apoptotic cells. The highest induction of apoptosis was seen in the doublet combination of Paclitaxel/Gallic acid (27.11%) which showed significant increase than single treatment with Paclitaxel and non-significant increase than the doublet combination Paclitaxel/Carboplatin. On the contrary, the doublet combination of Carboplatin/Gallic acid showed non-significant decrease in apoptotic population compared to single treatment with Carboplatin and the doublet combination Paclitaxel/Carboplatin.

### Evaluation of apoptosis by annexin V-FITC staining

**Fig. 3:**
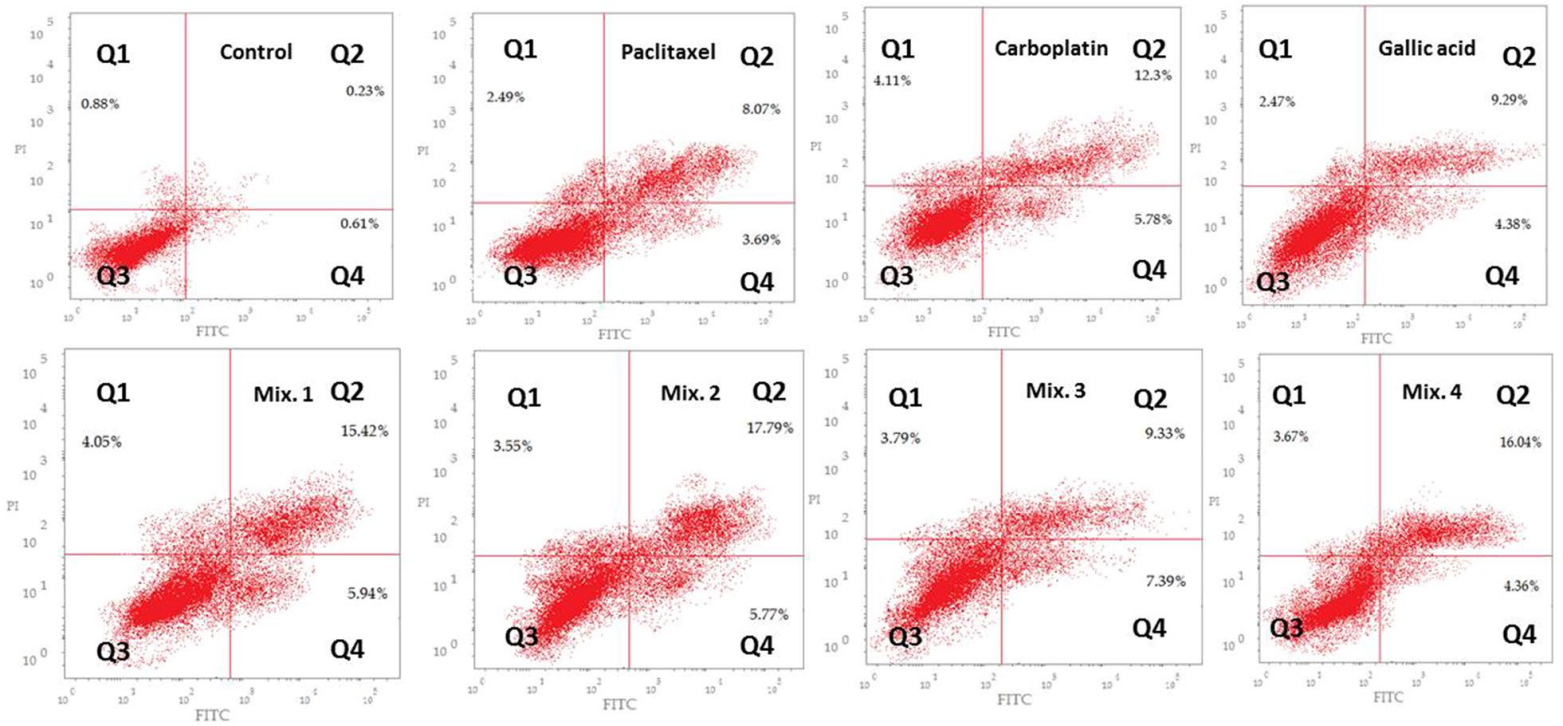
Dot plot representing four quadrant images observed by flow cytometric analysis. Q1: shows necrotic cells, Q2: shows later period apoptotic cells, Q3: shows normal cells and the Q4: shows early apoptotic cells

Since accumulation of cells at G2/M phase during cell cycle analysis was observed following treatment with Paclitaxel, Carboplatin, Gallic acid and the mentioned drug combinations, we were interested in quantifying the different types of apoptotic cells. In order to detect and quantify the apoptosis, Annexin V-FITC/PI double staining was used. Staining with Annexin V is typically used in conjunction with a vital dye such as PI for identification of early and late apoptotic cells. Viable cells with intact membranes exclude PI, whereas the membranes of dead and damaged cells are permeable to PI. Annexin V is capable of staining apoptotic cells as soon after initiating apoptosis; cells translocate the membrane phosphatidylserine (PS) from the inner face of the plasma membrane to the cell surface. Once on the cell surface, PS can be easily detected by staining with a fluorescent conjugate of Annexin Vas it has a high affinity for PS. Therefore, cells that are considered viable are both Annexin V and PI negative, while cells that are in early apoptosis are Annexin V positive and PI negative, and cells that are in late apoptosis or already dead are both Annexin V and PI positive. After HeLa cells were treated with selected doses of individual and combination drugs and stained with annexin V/PI, the cell cycle distribution was then detected by flow cytometry and results were recorded (Table 5) and represented as Dot plot graph representing four quadrant images (Fig. 3). Our results showed that both the doublet combination of Paclitaxel/Gallic acid (Mix. 2) and triplet combination of Paclitaxel/Carboplatin/Gallic acid (Mix. 4) showed the highest proportion of cells in late apoptotic stage (stained by Annexin V-FITC and PI). These results suggested synergistic or additive effect of Gallic acid with Paclitaxel and Paclitaxel/Carboplatin combination.

**Table 5:** Detection of different types of apoptotic cells induced in HeLa cells following treatment with different drugs using annexin VFITC/PI staining

ID	Total	Early apoptosis	Late apoptosis	Necrosis
Cont. (HeLa)	1.72	0.61	0.23	0.88
Paclitaxel	14.25	3.69	8.07	2.49
Carboplatin	22.19	5.78	12.3	4.11
Gallic acid	16.14	4.38	9.29	2.47
Mix. 1	25.41	5.94	15.42	4.05
Mix. 2	27.11	5.77	17.79	3.55
Mix. 3	20.51	7.39	9.33	3.79
Mix. 4	24.07	4.36	16.04	3.67

### Real time quantitative PCR (RT-qPCR) assay for some apoptotic markers

**Fig. 4:**
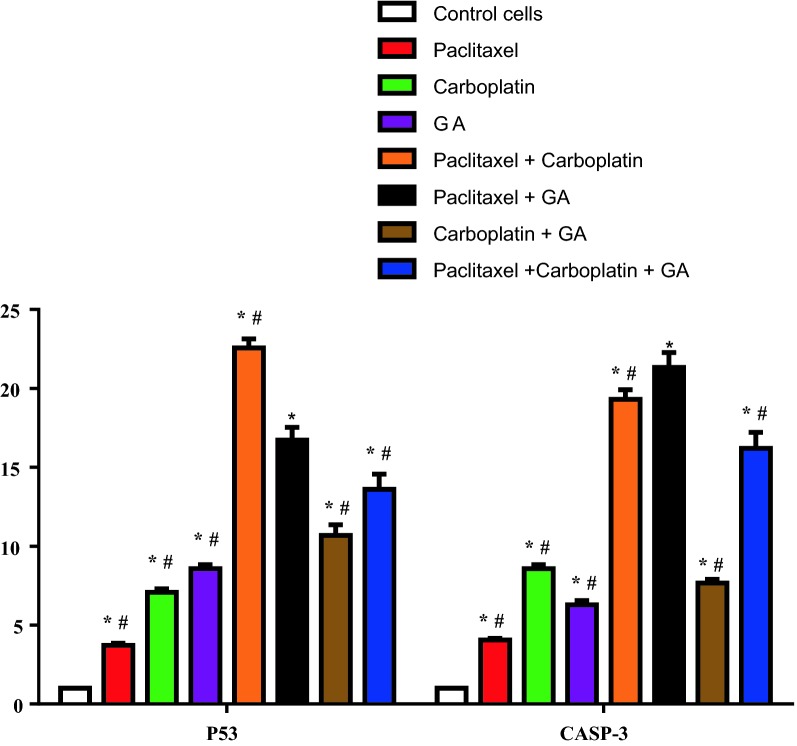
The expression of P53 and Caspase 3 by RT-qPCR. The results were represented as mean of fold changes ± SD (P < 0.0001). β-actin gene was used as a control gene. *Significant from HeLa group at P < 0.0001, ^#^significant from Paclitaxel + GA group at P < 0.0001

In order to analyze whether treatment with individual drugs or in combination for 24 h. affected genes controlling apoptosis including P53, Bcl-2, and Caspase 3, changes in the expression of these genes using quantitative real-time PCR were performed. The results are shown in Table 6 and Fig. 4. All cells treated with Paclitaxel, Carboplatin, Gallic acid and the mentioned drug combinations, showed significant increase in mRNA expression of P53 and Caspase 3 as compared to control HeLa cells, while BCl2 levels showed insignificant decrease than control HeLa cells. It was noticed that treatment of HeLa cell with Paclitaxel alone showed the least level of P53 (3.73 ± 0.14) and Caspase 3 (4.06 ± 0.1) among all treated group but upon addition of Gallic acid to Paclitaxel (Mix. 2), this combination therapy showed the highest level of Caspase3 (21.33 ± 0.95) and the second highest level of P53 (16.73 ± 0.81) preceded only by Paclitaxel/Carboplatin combination (Mix. 1) (22.56 ± 1.58). Paclitaxel/Gallic acid combination (Mix. 2) showed significant increase in P53 level as compared to all treated group except of Paclitaxel/Carboplatin combination (Mix. 1) at P < 0.0001. Paclitaxel/Gallic acid combination (Mix. 2) also showed significant increase in Caspase 3 level as compared to all treated group at P < 0.0001. These results suggested strong synergistic effect of Gallic acid with Paclitaxel.

**Table 6:** The expression of P53, Bcl-2, and Caspase 3 by RT-qPCR

Sample data	Results		
	Fold change		
ID	P53	bcl2	Casp3
Cont. HeLa	1	1	1
Paclitaxel	3.73 ± 0.14	0.38 ± 0.14	4.06 ± 0.1
Carboplatin	7.08 ± 0.22	0.24 ± .005	8.59 ± 0.25
Gallic acid	8.59 ± 0.25	0.28 ± 0.07	6.29 ± 0.27
Mix. 1	22.56 ± 1.58	0.28 ± 0.06	19.31 ± 0.61
Mix. 2	16.73 ± 0.81	0.28 ± 0.02	21.33 ± 0.95
Mix. 3	10.68 ± 68	0.22 ± 0.01	7.68 ± 0.23
Mix. 4	13.62 ± 0.96	0.31 ± 0.02	16.22 ± 1.01

The results were represented as mean of fold changes (2^ΔΔCT) ± SD. β-actin gene was used as a control gene

### Western blotting for some apoptotic markers

**Fig. 5:**
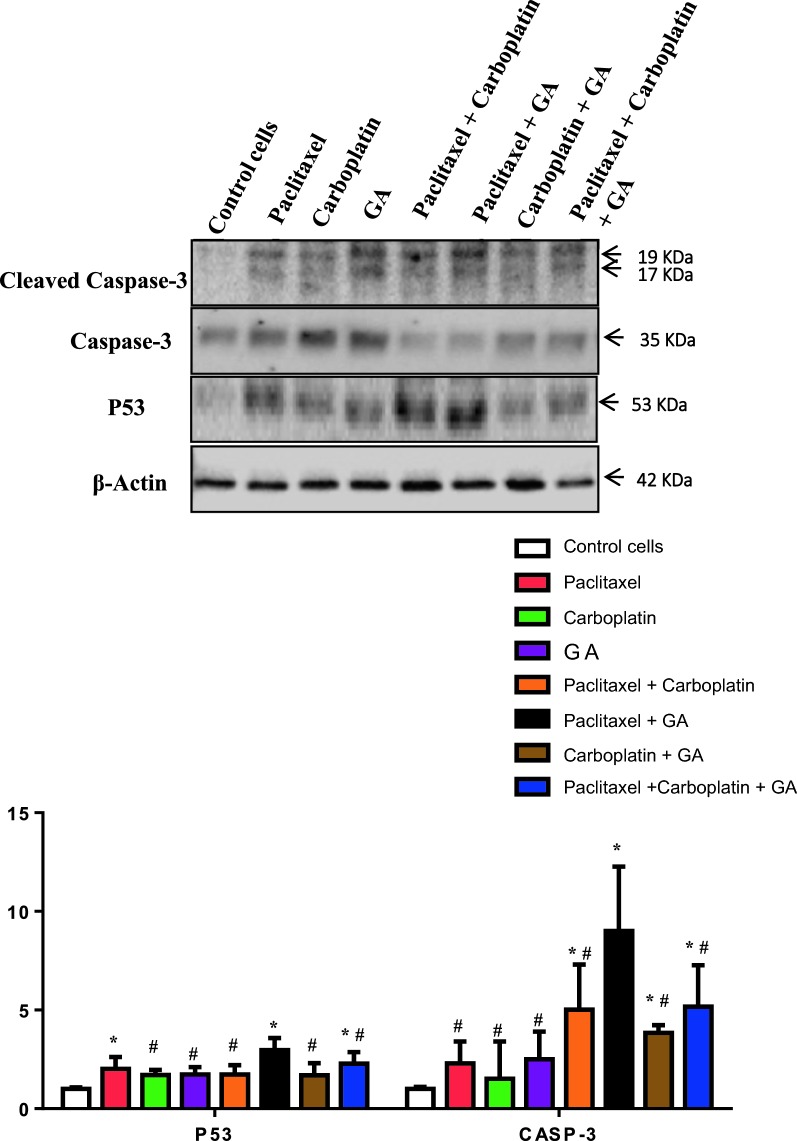
The immunoblotting of P53 and cleaved Caspase 3. Control group for both P53 and cleaved caspase-3/Caspase ratio and values were represented as mean ± SD of treated cells compared with Control cells (untreated cells) (P < 0.0001). The detected proteins were quantified and normalized to β-actin. *Significant from HeLa group at P < 0.01, ^#^significant from Paclitaxel + GA group at P < 0.05. The upper part of the figure illustrates immunoblot of P53, Cleaved Caspase-3/Caspase and β-actin from three separate experiments

**Fig. 6:**
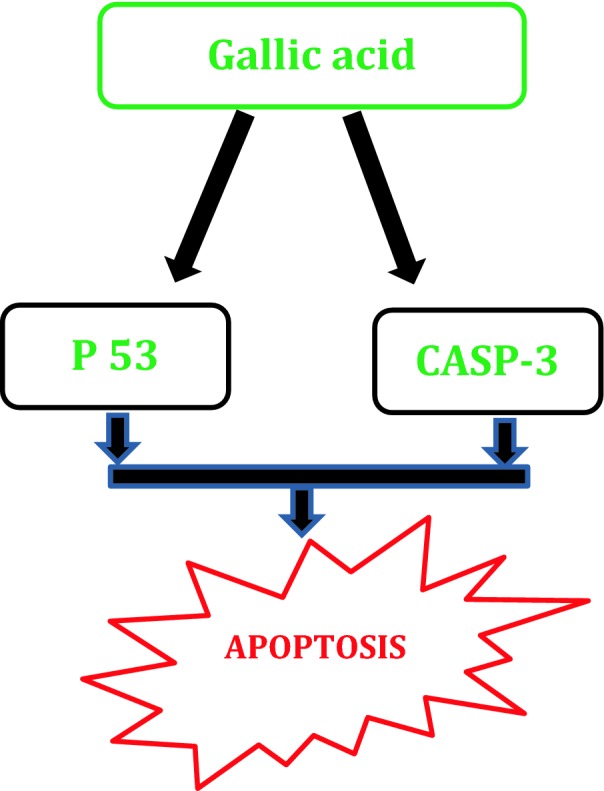
Schematic diagram showing the mechanism of action of Gallic acid in the induction of apoptosis in Hela cells

In an attempt to investigate the effect of different tested drugs on apoptotic pathway, level of P53 protein and ratio of Cleaved Caspase-3/Caspase 3 were assessed by western blotting (Fig. 5). The protein levels of both P53 and Caspase 3 in all tested drugs were significantly increased as compared to control HeLa cells. Paclitaxel/Gallic acid combination (Mix. 2) showed significant increase in P53 and Caspase 3 levels as compared to all groups. These results suggests that Paclitaxel/Gallic acid combination has the highest apoptotic effect as evidenced by its induction of both p53, a tumor suppressor protein that regulates apoptosis, and Caspase 3, a key enzyme in the execution of apoptosis, (Fig. 6). Taken together, Paclitaxel/Gallic acid combination could be an effective and safer alternative for Paclitaxel/Carboplatin combination.

## Discussion

Although chemotherapeutic drugs are very effective, severe side effects and development of drug resistance limits the use of these drugs. The use of natural products with anticancer activity may help to partially overcome these issues. In the present study, we investigated the ability of Gallic acid, to potentiate the anti-cancer effects of Paclitaxel, Carboplatin and Paclitaxel/Carboplatin combination in human HeLa cells by performing MTT assay, cell cycle analysis, RT-PCR assay for P53, Bcl-2, and Caspase 3 genes and Western blot analysis for P53 and Caspase 3. Our results revealed that the most promising combination was Gallic acid with Paclitaxel as it showed the highest cytotoxic effect by MTT assay, the highest induction of apoptosis by cell cycle analysis and significant elevation in P53 and Caspase 3 levels.

A number of previous studies investigated the cytotoxic effect of GA in various cell lines and showed that the IC50 for Calu-6 (human, Caucasian, lung, adenocarcinoma), A 549 (adenocarcinomic human alveolar basal epithelial cells), HeLa (human epithelial carcinoma), and (HUVECs) Human umbilical vein endothelial cells were 10–50, 100–200, 80, 400 µM, respectively [28–31]. These results suggest that Gallic acid induced cell death in tumour cells with a relatively high selectivity. In the present study, Gallic acid treatment decreased the growth of HeLa cells in a dose-dependent manner, with the IC50 value of ~ 11.5 µM at 24 h. The cytotoxic effect of Gallic acid may be explained by its pro-oxidant property which has been recognized as apoptosis inducer in different cancer cell lines including HeLa cells [32]. The difference in IC50 of Gallic acid in HeLa cell seen in our study can be explained by the fact that although MTT assay has been used for 30 years in cancer research [33–35], it rarely yielded a consistent IC50 value for a given chemical compound against a given cancer cell line. According to He et al. [36], this issue was attributed to differences among the manufacturers and formulae used by different laboratories, even within the same laboratory, the MTT assay results gives variable IC50 values among different staff researchers and between different experimental repeats performed by the same researcher. This inconsistency can be explained by variation in control wells, which are used as a basis for the calculations of IC50 values, these variations depend on the initial cell seeding density and the proliferation potential of the cell line [36].

In the present study, flow cytometric analysis showed that Gallic acid caused G2/M phase cell cycle arrest. Subsequently, the apoptotic sub-G1 phase increased obviously, suggesting the occurrence of sequential events of cell cycle arrest followed by apoptosis. Gallic acid was found to induce apoptosis in different in vitro [30] and in vivo studies [37–39].

The ability of Gallic acid to induce G2/M arrest was also reported by Ou et al. [40] who explained this effect by alterations in cell cycle regulators in a human bladder transitional carcinoma cell line. The regulation of cell cycle is controlled by a family of cyclin/cyclin-dependent kinase (CDK) complexes and the CDK inhibitors (CDKIs) [41]. G2/M transition is regulated mainly by the sequential activation and deactivation of CDK-regulatory proteins and cyclin complexes. Cdc2 is also known as Cdk1, which initially forms a complex with Cyclin B1 to drive the cell from G2 to M phase [42]. Ou et al. [40] suggested that the anticancer effect of Gallic acid seems to be based on activation of ataxia telangiectasia-mutated (ATM)/checkpoint kinase 2 (Chk2)-which cause phosphorylation and activation of cdc25c, a phosphatase that activates the Cdk1/cyclin B1 kinase complex, leading to accumulation of cdc2 in Tyr-15 phosphorylated inactive form and consequently cell cycle arrest and apoptosis occur.

Apoptosis is a complex process that proceeds through at least two main pathways (extrinsic and intrinsic), each of which can be regulated at multiple levels. The intrinsic pathway centers on the mitochondria, the release of proapoptotic factors from the mitochondria, such as cytochrome c, into the cytoplasm promotes the activation of initiator caspase-9, which in turn activates effector caspases such as caspase-3 or -7. The extrinsic pathway relies on the activation of death receptors from the tumour necrosis factor (TNF) receptor family that promote the recruitment and activation of initiator caspase-8 via adaptor proteins such as Fas-associated death domain (FADD) or TNFRSF1A-associated via death domain (TRADD) [43]. Major regulators of the intrinsic pathway are the pro- and anti-death members of the Bcl-2 family [44]. These proteins reside at, or translocate to the mitochondria, controlling the release of p53, a major tumour suppressor, serves as a regulator of the apoptotic process that can modulate key control points in both the extrinsic and intrinsic pathways [45].

In our study, Gallic acid induced apoptosis as manifested by the results of both MTT assay and flow cytometric analysis. In order to understand the role of Gallic acid in induction of apoptosis, P53, Bcl-2, and Caspase 3 expression was detected by RT-PCR assay and Western blot analysis.

In our study, Paclitaxel/Gallic acid combination showed the second highest level of P53 preceded only by Paclitaxel/Carboplatin combination. P53 is necessary to maintain a G2 arrest following DNA damage, since tumor cells lacking this proteins enter into mitosis with accelerated kinetics. The mechanism of p53-dependent G2 arrest involves an initial inhibition of cyclin B1/Cdc2 activity by p21 and a subsequent reduction of cyclin B1 and Cdc2 protein levels [46]. The ability of Gallic acid to up regulate p53 seen in our study is in agreement with Wang et al. [47] who revealed that Gallic acid exhibited its anticancer effect on human SCLC H446 cells via the ROS-dependent mitochondrial apoptotic pathway.

Caspase 3 is one of key enzymes to play a pivotal role in the terminal execution phase of apoptosis [48]. In Our study, Paclitaxel/Gallic acid combination showed the highest level of Caspase 3. The involvement of different Caspases in GA-induced apoptosis in HeLa cells, including Caspase 3, was reported by You et al. [28] who found that treatment of GA-treated HeLa cells with various Caspase inhibitors resulted in significantly but not completely prevention of apoptosis and decreased dead cell percentage.

The potentiating effect of co-treatment of Gallic acid with Paclitaxel seen in our study, is in agreement with Sánchez-Carranza et al. [49] who reported that the cytostatic action of Paclitaxel is increased in the human ovarian carcinoma A2780 cells (drug sensitive) and A2780AD cells (multi-drug-resistant ovarian cancer) models via co-treatment with Gallic acid.

Sánchez-Carranza et al. explained this result by the ability of Gallic acid to potentiate the Paclitaxel-induced G2/M phase arrest. Using the drug-resistant cell line, they also demonstrated that proliferation inhibition and G2/M phase arrest are mediated by Gallic acid-provoked reactive oxygen species overproduction, and by reactive oxygen species-mediated inhibition of Paclitaxel-provoked extracellular signal-regulated kinases (ERKs) activation [50]. ERK is a subfamily of mitogen-activated protein kinases (MAPKs) that control a vast array of physiological processes. The extracellular signal-regulated kinases (ERKs) function in the control of cell division, and inhibitors of these enzymes are being explored as anticancer agents [40].

## Conclusion

The present study suggests that the combination of Paclitaxel and Gallic acid may be a promising protocol for treatment of cervical cancer and it could be a possible replacement of the current commonly used regimen Paclitaxel/Carboplatin. The Paclitaxel/Gallic acid combination offers a number of advantages over existing protocols such as higher efficacy, lower dose and less side effects. More detailed mechanistic and efficacy studies are warranted in cell culture, animal models and finally clinical trials to evaluate Paclitaxel/Gallic acid combination therapy.

## Data Availability

The datasets used and/or analysed during the current study are available from the corresponding author upon reasonable request.
